# Blood pressure and acute kidney injury

**DOI:** 10.1186/s13054-017-1611-7

**Published:** 2017-02-10

**Authors:** Ryota Sato, Sarah Kyuragi Luthe, Michitaka Nasu

**Affiliations:** 1 Department of Emergency and Critical Care Medicine, Urasoe General Hospital, 4-16-1, Iso, Urasoe, Okinawa 901-2131 Japan; 2 Department of Anesthesia, Urasoe General Hospital, Okinawa, Japan

**Keywords:** Acute kidney injury, Central venous pressure, Mean arterial pressure, Mean perfusion pressure, Diastolic perfusion pressure

## Abstract

Maintaining the optimal blood pressure is an important aspect of preventing acute kidney injury (AKI), especially for vasopressor-dependent patients. Although mean arterial pressure (MAP) has played an important role in previous trials for prevention of AKI, there is little evidence that MAP actually reflects organ perfusion. In fact, several studies have suggested that perfusion pressure, including diastolic perfusion pressure and mean perfusion pressure (MPP) and calculated with central venous pressure (CVP), may be more useful than the widely used MAP to help prevent AKI. This emphasizes the importance of maintaining diastolic arterial pressure and avoiding elevation of CVP to prevent AKI in patients with sepsis or invasive surgery. To achieve this, further investigation regarding titrated fluid therapy and vasopressors is warranted.

Blood pressure has been considered to be essential for organ perfusion. Therefore, maintaining the optimal blood pressure is an important aspect of preventing acute kidney injury (AKI), especially for vasopressor-dependent patients. Mean arterial pressure (MAP) is widely used as an index for the optimal blood pressure.

However, although the purpose of maintaining MAP is to provide adequate organ perfusion, there is little evidence that MAP actually reflects organ perfusion. Recently, perfusion pressure has been considered as an indicator in the context of preventing AKI. In terms of perfusion pressure, diastolic perfusion pressure (DPP) and mean perfusion pressure (MPP) should be pointed out. These are defined as: DPP = Diastolic arterial pressure (DAP) − Central venous pressure (CVP); and MPP = MAP − CVP. A recent observational study revealed that lower DAP and higher CVP were associated with septic AKI, while MAP was not [1]. Another observational study reported that decreased MPP was associated with AKI while MAP was not, suggesting that only CVP may be associated with septic AKI [2]. Furthermore, a recent study of post-cardiac surgery patients showed an association between decreased DAP, MPP, and DPP, but not MAP, and AKI. The decreased DPP was caused by elevation of CVP in 23.8% of the patients and by decreased DAP in 76.2% [3].

Although aggressive fluid resuscitation is believed to protect the kidney, this may contribute to the elevation of CVP and consequently AKI as demonstrated above. Previous studies have reported an association between fluid overload and AKI [4]. Additionally, the change in DAP is believed to reflect the alteration of vascular tone. Therefore, since decreased DAP is associated with AKI, vasopressors may be effective for the prevention of AKI [5].

From these perspectives, it is important to maintain DAP and avoid the elevation of CVP to prevent AKI in patients with sepsis or after invasive surgery. To achieve this, further investigation regarding titrated fluid therapy and vasopressors is warranted.

## Abbreviations

AKI: Acute kidney injury
CVP: Central venous pressure
DAP: Diastolic arterial pressure
DPP: Diastolic perfusion pressure
MAP: Mean arterial pressure
MPP: Mean perfusion pressure
